# High intensity endurance training is associated with better quality of life, but not with improved cognitive functions in elderly marathon runners

**DOI:** 10.1038/s41598-019-41010-w

**Published:** 2019-03-15

**Authors:** D. Batmyagmar, M. Kundi, E. Ponocny-Seliger, I. Lukas, J. Lehrner, H. Haslacher, R. Winker

**Affiliations:** 1 Centre for Public Health, Medical University of Vienna, Vienna, Austria; 2 Empirical Research, Vienna, Austria; 3 Health and Prevention Centre, Sanatorium Hera, Vienna, Austria; 4 Department of Neurology, Medical University of Vienna, Vienna, Austria; 5 Department of Laboratory Medicine, Medical University of Vienna, Vienna, Austria

## Abstract

Impairment of cognitive functions in advanced age leads to a reduced quality of life and impaired ability to perform everyday tasks. The positive impact of physical exercise on the quality of life and well-being, also at a later age, is well established. However, the effect of endurance exercises, including long distance running and cycling, on cognitive function and mental health within the elderly population has still to be elucidated. To this end, elderly active marathoners (N = 50) aged over 60 years and non-athlete controls (N = 49) were followed for four years. Cognitive function was assessed using the CERAD test battery. In addition, the Short Form Health Survey (SF-36) was applied to assess self-reported physical, mental, and emotional health. Except for age, sex and education-corrected z-values of the test “Word list recall”, with marathon runners showing a decline compared to an improvement in controls (p < 0.05), there was no statistically significant difference in time trend between groups. In contrast, concerning self-reported health, scores in all eight domains of the SF-36 remained stable over time and, in nearly all of them, marathon runners showed higher self-reported health than controls. The results indicated that extensive endurance exercise is associated with improved subjective health but does not lead to better scores in cognitive performance tests in elderly persons.

## Introduction

There is general consensus that moderate physical exercise is a healthy undertaking at all ages, including the elderly. As the population in the industrialized world continues to age, and cognitive functions slowly decline at an advanced age, this raises the question of whether physical exercise might combat cognition decay. Although light exercises, including yoga and aerobics, have been shown to improve performance in cognitive tests over the short term^1–3^, the question of whether endurance sports in the elderly lead to similar improvements in the long term has not yet been addressed. Hence, in light of the importance of guiding the elderly population towards a lifestyle that would increase the likelihood of maintaining cognitive performance through advanced age, there is a need to assess whether more intensive exercise would represent a way to conserve cognitive function.

The aforementioned demographic change observed in industrialized countries is mainly due to improvements in the quality of healthcare and general living standards. According to predictions by the World Health Organization, the population aged over 60 years will almost double from 12% in 2015 to 22% in 2050^4^. A similar demographic change is anticipated in Austria: the proportion of the population aged 65 years and older has been estimated to increase from 18% in 2013 to 24% in 2030. In 2013, the average life expectancy in Austria for males was 78.45 years and 83.56 years for females and was expected to increase by 2030 to 80.45 years for males and 85.53 years for females^5^.

Accompanied senescence of body tissues and functions, known to be associated with a number of chronic age-related diseases, including metabolic syndromes, osteoporosis, cardiovascular diseases, and cancer also has an impact on cognitive function and may result in severe cognitive impairment and dementia^6^. Chronic diseases impose huge burdens for individuals but also on the social welfare system and cause increased healthcare expenditures^7,8^. In 2016, nearly 44 million people worldwide were living with dementia^9^. Associated healthcare costs were US$ 604 billion in 2010, increased significantly by 35% to US$ 818 billion in 2015 and are expected to keep rising in the future^10^. Therefore, WHO declared in 2012 that dementia is a public health priority^11^ and pointed out that primary prevention would be the most effective and affordable way to reduce the onset of dementia^12^.

There is a continuing debate about how physical and mental health can be maintained at an advanced age, and which interventions might be cost-effective to prevent or delay age-related physical and mental degradation. It is well known that low to moderate intensity physical activity is beneficial to overall health and especially regarding cognitive function^13–15^. In this regard, there is growing evidence that physical exercising is neuroprotective and enhances immune competence^16,17^. Besides that, physical activity ameliorates depressive symptoms, even if genetic susceptibility is present^18,19^, and improves subjective well-being and quality of life^20,21^ Accordingly, general recommendations on physical activity for elderly above the age of 65 years propose at least 150 min/week of moderate-intensity aerobic exercise, if a substantial health benefit is desired^22^. The underlying mechanisms are not yet fully understood, although different modes of action were proposed. These include the induction of neuro- and synaptogenesis, the release of neurotrophins^23^ and also the modulation of gut microbiota^24^.

Likewise, there is very little knowledge about how high intensity endurance training might affect cognitive functions and well-being in the elderly. This form of training includes high intensity, regular, and long-term physical activity such as long distance running and cycling. So far, some authors reported a positive effect of vigorous activity on mental health^21,25^. Here, we aimed to increase the spare existing knowledge on the interplay between vigorous physical activity, cognitive functions and mood by studying a cohort of elderly marathon runners and long-distance cyclists, as well as a control group with low physical activity during almost four years in a prospective, controlled observational trial. Both groups were assessed regarding changes in cognitive functions as measured by the German Plus-version of neuropsychological test battery designed by the Consortium to Establish a Registry for Alzheimer’s disease (CERAD). This test battery is a reliable measure to identify patients with any type of dementia and covers a broad range of cognitive domains, including language, memory, and other executive functions, which are amongst the earliest functions impaired in Alzheimer’s disease)^26,27^. Moreover, the battery provides standard values corrected for age and years of education, enabling the calculation of individual deviations from the normal range (“z-values”)^26^.

## Results

### Cohort description

**Figure 1:**
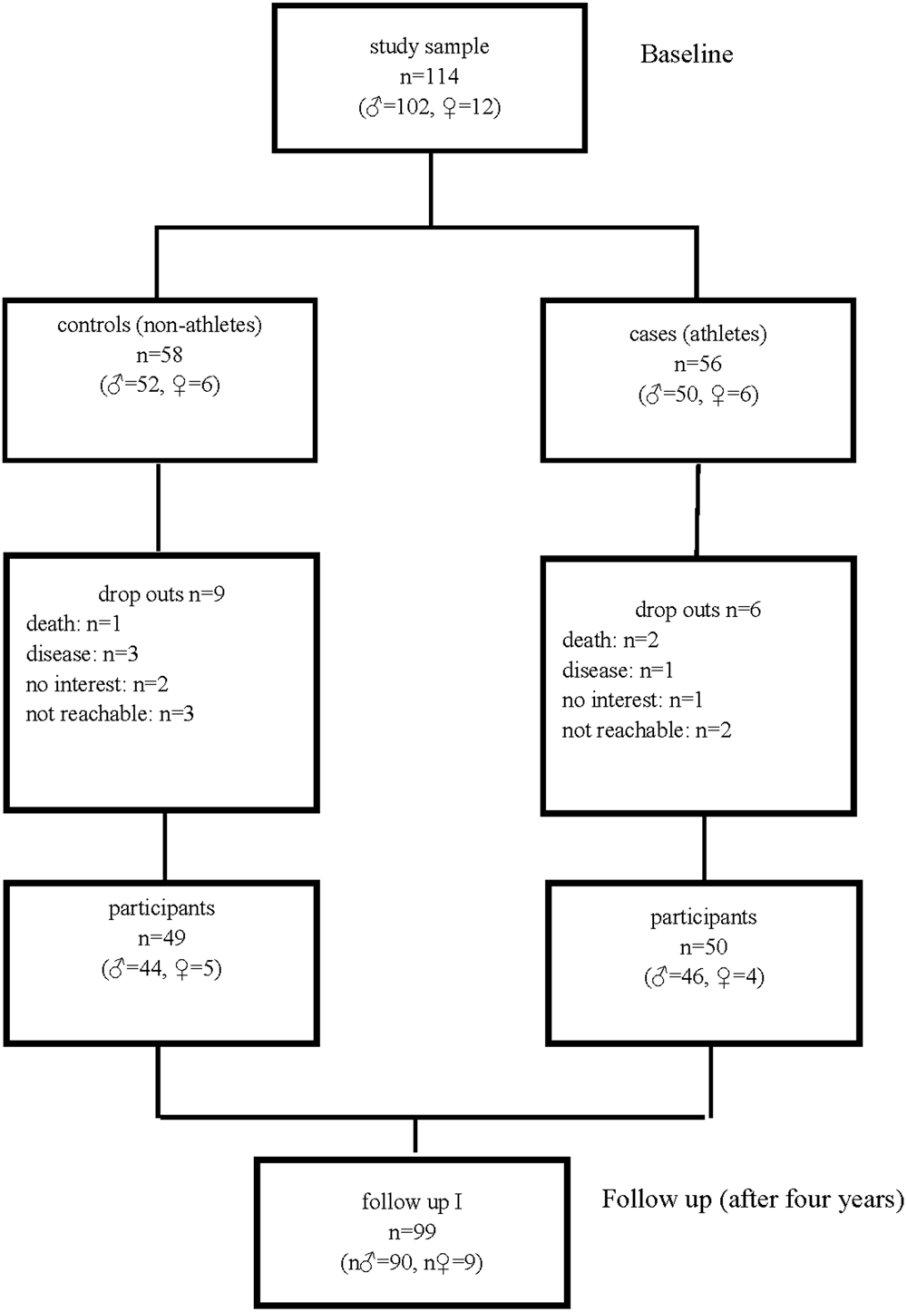
Study flow chart.

Overall, 99 participants (50 athletes, 49 controls) were included in the present analysis comprising 86% of the initially enrolled individuals (Fig. 1). The mean age at baseline was 66 ± 4 years for the control group and 66 ± 5 years for the athletes. No statistically significant differences were observed for gender and years of education (original matching variables) and family history of Alzheimer’s disease, current medication, and alcohol consumption. Body mass index (BMI) differed significantly (p_BH_ <0.0001) between the two groups (controls 27.6 ± 4.9 and athletes 23.7 ± 2.6 kg/m²). A significantly lower number of athletes reported themselves as smokers, or to suffer from a relevant disease (all p_BH_ <0.05 Table 1).

**Table 1:** General characteristics of the study groups (follow up I in 2012). Mean (SD), or n (%).

Characteristic		Controls N = 49	Athletes N = 50	P-value
		Mean ± SD or N (%)		
Age (years)		66 ± 4	66 ± 5	0.957^ns^
Sex	*Female*	5 (10%)	4 (8%)	0.703^ns^
	*Male*	44 (90%)	46 (92%)	
Education (years)		12 ± 4	11 ± 4	0.359^ns^
Body mass index (BMI, kg/m^2^)		27.6 ± 4.9	23.7 ± 2.6	<0.0001***
Smoker	*No*	22 (45%)	39(78%)	0.003*
	*Yes*	7 (14%)	2 (4%)	
	*Ex smoker*	20 (41%)	9 (18%)	
Medication	*No*	14 (29%)	26 (52%)	0.018^ns^
	*Yes*	35 (71%)	24 (48%)	
Alzheimer in the family	*No*	43 (88%)	41 (82%)	0.425^ns^
	*Yes*	6 (12%)	9 (18%)	
Relevant disease^a^	*No*	29 (59%)	42 (83%)	0.006*
	*Yes*	20 (41%)	8 (16%)	
Alcohol consumption (ml/day)		13 ± 15	10 ± 12	0.282^ns^

SD-Standard deviation.

^a^Diseases which is not affected Central Nervous System functions, including hypertension, diabetes mellitus II, pulmonary disease, rheumatoid arthritis, hyperlipidemia, prostate cancer.

^ns^Non-significant after Benjamini/Hochberg correction, *<0.05 after Benjamini/Hochberg correction, ***<0.001 after Benjamini/Hochberg correction.

### Relative Physical performance

General physical performance of the participants is given in Table 2. Athletes performed almost 50% better than individuals of the control group, both at baseline and during the follow-up exam in 2012 (p < 0.001, Table 2). Also, the decline in absolute physical performance was less expressed in athletes than in controls, although this observation lost statistical significance after correction for multiple hypothesis testing.

**Table 2:** Treadmill test.

Variable	Controls N = 49		Athletes N = 50		Within-subjects P-value	Between-subjects P-value	Interaction P-value
	Baseline Mean ± SD	Follow-up Mean ± SD	Baseline Mean ± SD	Follow-up Mean ± SD			
Absolute performance (W)	146 ± 37	130 ± 35	205 ± 37	199 ± 35	<0.0001**	<0.00001***	0.038^ns^
Relative performance (%)	100 ± 27	89 ± 23	154 ± 27	148 ± 23	<0.00001***	<0.00001***	0.107^ns^

Within-subjects: Comparison of performances between baseline and follow-up in a general linear model with repeated-measurements design.

Between-subjects: Comparison o performances between athletes and controls in a general linear model with repeated-measurements design.

Interaction: Comparison of performance developments between athletes and controls in a general linear model with repeated-measurements design.

SD-standard deviation.

^ns^Non-significant after Benjamini/Hochberg correction, **<0.01 after Benjamini/Hochberg correction, ***<0.001 after Benjamini/Hochberg correction.

### Cognitive performance is not better in athletes than in non-athletes

When analyzing z-scores of the subtests of the CERAD Plus neuropsychological test battery using general linear models, it seemed that both athletes and controls improved in the S-word test assessing phonemic verbal fluency (p = 0.011). In contrast, z-scores in constructional praxis declined in both groups between baseline and follow-up measurements (p = 0.045), whereby athletes presented with a higher z-score than controls (p = 0.033). However, all three results did not maintain statistical significance after correction according to Benjamini and Hochberg. The only significant difference in temporal developments of z-scores between athletes and controls were registered for the subtest “Word list recall” assessing episodic memory. In this subscale, values of controls improved, while z-scores of athletes slightly deteriorated (athletes: z = −0.31 ± 1.24 vs. −0.52 ± 1.30, controls: −0.66 ± 1.24 vs. −0.20 ± 1.29, p_BH_ <0.05). Detailed numbers are given in Table 3.

**Table 3:** Means (±SD) for scores of the Consortium to Establish a Registry for Alzheimer’s disease test battery (CERAD) and results of two-factor ANOVA for effects of time, groups, and interaction, controlled for the habit of social communication

Subtest scores (range of values)	Group				Within-subjects		Between-subjects		Interaction	
	Controls (N = 49)		Athletes (N = 50)		F	P-value	F	P-value	F	P-value
	Baseline Mean ± SD	Follow-up Mean ± SD	Baseline Mean ± SD	Follow-up Mean ± SD						
Global cognitive functions										
Mini mental state exam^↑^	−0.98 ± 1,31	−0.56 ± 1.30	−0.64 ± 1.31	−0.26 ± 1.30	1.25	0.267^ns^	2.12	0.149^ns^	0.01	0.914^ns^
Language Functions										
Boston Naming test^↑^	0.37 ± 0.79	0.53 ± 0.71	0.30 ± 0.79	0.59 ± 0.10	3.36	0.070^ns^	<0.01	0.963^ns^	0.72	0.399^ns^
Verbal fluency^a↑^	0.98 ± 1.03	0.75 ± 1.24	1.00 ± 1.03	0.57 ± 1.24	2.51	0.117 ^ns^	0.14	0.712 ^ns^	0.91	0.344 ^ns^
S- words^↑^	0.85 ± 1.05	1.28 ± 1.23	0.55 ± 1.05	0.86 ± 1.23	6.65	0.011 ^ns^	2.79	0.098 ^ns^	0.37	0.544 ^ns^
Visuo-construction										
Constructional praxis^↑^	0.58 ± 0.58	0.28 ± 0.64	0.70 ± 0.57	0.61 ± 0.64	4.15	0.045 ^ns^	4.68	0.033 ^ns^	2.33	0.130 ^ns^
Other execute functions										
Trail making test B↓	−0.38 ± 0.88	−0.13 ± 1.36	−0.53 ± 0.88	−0.17 ± 1.37	2.67	0.106 ^ns^	0.267	0.607 ^ns^	0.11	0.739 ^ns^
Trail making test A/B^b^	0.08 ± 0.97	1.77 ± 6.73	−0.04 ± 0.97	0.23 ± 6.73	0.80	0.373 ^ns^	1.45	0.232 ^ns^	1.04	0.311 ^ns^
Episodic memory										
Word list encoding, total^↑^	−0.64 ± 1.12	0.36 ± 1.41	0.04 ± 1.13	−0.02 ± 1.41	0.16	0.687 ^ns^	0.37	0.545 ^ns^	3.48	0.065 ^ns^
Word list recall^↑^	−*0.66* ± *1.24*	−*0.20* ± *1.29*	−*0.31* ± *1.24*	−*0.52* ± *1.30*	0.84	0.361 ^ns^	0.01	0.938 ^ns^	7.66	0.007*
Word list discrimination^↑^	−0.07 ± 1.13	0.19 ± 1.02	<0.01 ± 1.13	−0.23 ± 1.02	0.02	0.879 ^ns^	0.95	0.331 ^ns^	3.47	0.066 ^ns^
Constructional praxis recall^↑^	0.55 ± 0.84	0.24 ± 1.08	0.51 ± 0.85	0.36 ± 1.09	0.03	0.868 ^ns^	0.06	0.804 ^ns^	0.44	0.507 ^ns^
Attention and processing speed										
Trail making test A↓	−0.61 ± 0.67	−0.16 ± 1.06	−0.63 ± 0.67	−0.37 ± 1.05	2.03	0.158 ^ns^	0.55	0.460 ^ns^	1.09	0.300 ^ns^

Within-subjects: Comparison of z-values between baseline and follow-up in a general linear model with repeated-measurements design.

Between-subjects: Comparison of z-values between athletes and controls in a general linear model with repeated-measurements design.

Interaction: Comparison of z-value developments between athletes and controls in a general linear model with repeated-measurements design.

^a^Animal names, ^b^Trail making test A/B (trail making B/trail making A), ↓ lower scores indicate better results, ^↑^ higher scores indicate better results, italics: increase/decrease dependent on group.

^ns^Non-significant after Benjamini/Hochberg correction, * < 0.05 after Benjamini/Hochberg correction.

Hence, it appeared that athletes were not better than non-athletes in neuropsychological surrogates of all cognitive domains. On the contrary, controls presented with a significant improvement of a memory-related subtest, while athletes declined. In a next step, we aimed to assess whether both groups differed in subjectively perceived health and wellbeing.

### Self-reported health is higher in endurance athletes compared to non-athletes

To assess whether endurance sports had any impact on subjective well-being, athletes and controls were asked to self-evaluate their health by the SF-36 at both time points. In general, athletes evaluated their health as better than non-athletes in the following categories: Physical functioning (p_BH_ <0.001), bodily pain (p_BH_ <0.05), physical role functioning (p_BH_ <0.01), general health perceptions (p_BH_ <0.01), vitality (p_BH_ <0.01), social functioning (p_BH_ <0.01), and emotional role functioning (p_BH_ <0.05). Solely the differences in mental health perception (p = 0.016) were no longer statistically significant after p-value correction (Table 4).

**Table 4:** Self-reported health and well-being questionnaire with eight dimensions (SF-36).

SF-36 Dimension*	Group				Within-subjects		Between-subjects		Interaction	
	Controls (N = 49)		Athletes (N = 50)		F	P-value	F	P-value	F	P-value
	Baseline Mean ± SD	Follow-up Mean ± SD	Baseline Mean ± SD	Follow-up Mean ± SD						
Physical functioning	87 ± 10	84 ± 12	97 ± 10	97 ± 12	2.91	0.091 ^ns^	32.91	<0.00001***	1.32	0.252 ^ns^
Bodily pain	76 ± 23	76 ± 20	89 ± 23	87 ± 20	0.090	0.765 ^ns^	8.90	0.004*	0.181	0.671 ^ns^
Physical role functioning	80 ± 25	79 ± 25	97 ± 25	96 ± 250	0.116	0.734 ^ns^	15.02	<0.001**	0.005	0.943 ^ns^
General health perceptions	81 ± 18	68 ± 15	81 ± 18	80 ± 15	2.01	0.160 ^ns^	14.21	<0.001**	0.624	0.431 ^ns^
Vitality	69 ± 14	69 ± 16	80 ± 14	79 ± 16	0.445	0.506 ^ns^	13.37	<0.001**	0.412	0.523 ^ns^
Social functioning	87 ± 14	90 ± 15	97 ± 14	97 ± 15	0.997	0.321 ^ns^	11.30	0.001**	0.714	0.400 ^ns^
Emotional role functioning	84 ± 24	82 ± 28	98 ± 24	96 ± 28	0.385	0.537 ^ns^	1.42	0.002*	0.000	0.995 ^ns^
Mental health	78 ± 14	77 ± 13	84 ± 14	84 ± 13	0.106	0.746 ^ns^	6.07	0.016 ^ns^	0.124	0.725 ^ns^

*The SF-36 dimensions are scored on a 0 (poor) to 100 (good health) scale Within-subjects: Comparison of scores between baseline and follow-up in a general linear model with repeated-measurements design Between-subjects: Comparison of scores between athletes and controls in a general linear model with repeated-measurements design Interaction: Comparison of score developments between athletes and controls in a general linear model with repeated-measurements design SD-Standard deviation.

^ns^Non-significant after Benjamini/Hochberg correction, * < 0.05 after Benjamini/Hochberg correction, ** < 0.01 after Benjamini/Hochberg correction, *** < 0.001 after Benjamini/Hochberg correction.

Our results indicated that high intensity endurance training is associated with better subjective health perception and wellbeing.

## Discussion

In this study, we investigated the effect of high intensity endurance training on cognitive and physical performance as well as well-being in healthy older people. We found no strong evidence that long-term, high intensity endurance training reduced cognitive impairment in later age. However, elderly athletes reported a significantly better quality of life.

Previous interventional and cohort studies had investigated the protective effect of physical activity on cognitive performance in the elderly. Most of the published interventional studies focused on light to moderate sports activities, including walking, gymnastics, yoga, and aerobic exercises^28,29^. The results of these earlier studies indicated that the dosage and intensity of the training might be important for preventing the deterioration of cognitive function. The World Health Organization recommends that being active is better than remaining sedentary. Even a small amount of movement, including daily activities and walking, can have a positive impact on cognitive functioning and preventing its impairment in later life^30,31^. It was also claimed that moderate- and high intensity-persistence exercise programs have an equally beneficial effect on cognitive function^32,33^. In contrast, Kimura K *et al*. found that short-term (12 weeks) training has a positive effect on health-related quality of life (HRQOL), but did not affect executive functions^34^.

In our study, at the four-year follow-up stage, both the athletes as well as the non-athlete controls scored lower in four out of 15 tasks of the test battery designed by the Consortium to Establish a Registry for Alzheimer’s disease (data not shown), however, there was no statistically significant decline, when z-values (corrected for sex, age and years of education) were compared instead of raw scores. Hence, the observed cognitive decline might be related to the natural aging process.

In detail, during follow-up, Word List Recall appeared improved in controls, but deteriorated slightly in athletes. In both groups, performance in the S-word test increased. A slight decline was also noted for Constructional praxis, but this was likely due to a regression to the mean effect because, in 2008, performance was at the maximum for almost all participants. However, both developments (S-words improvement and decline of Constructional praxis) did not remain statistically significant after correction for multiple hypothesis testing. The same was true for the apparent dominance of athletes in Constructional praxis, which lost statistical significance as well.

In contrast, athletes reported better physical and cognitive health and well-being in nearly all subscales of the SF-36, which is accompanied by significantly lower depressive symptoms, as reported before^18,19,35^. This is in-line with previous findings among all age groups and types of exercise; in law students, e.g., the degree of mental distress correlated well with their levels of exercise^36^. For sedentary older adults, it was reported that Yoga interventions markedly improved physical function and well-being^37^. As underlying mechanisms, neuromodulatory effects were proposed. In this regard, it was demonstrated that exercise might stem the reduction in hippocampal neurogenesis as it is seen in depressive patients^38^. In fact, exercise therapy has meanwhile become a standard treatment for mild-to-moderate depression^39^, especially in patients without physical co-morbidity^40^.

Our present results appear at first glance to be counterintuitive, as one would assume that endurance athletes would enjoy not only better health but also slower cognitive decline, which was apparently not evident here. This is indeed in line with previous observations^41,42^. We hypothesize that daily high intensity endurance training requires a great deal of time, which is no longer available for social contact. There is a considerable volume of literature demonstrating a relationship between cognitive function and social interaction. These studies emphasize that an active social life in older age is essential for maintaining healthy cognitive function^43,44^. For instance, social engagements with friends offer protection against cognitive decline in women^43^, and a higher social activity is associated with better cognitive function^44^.

An additional potential explanation for the observed reduction in athletes’ cognitive performance might be attributed to high amounts of free radicals produced during high intensity endurance training, which at a senior age might not be as effectively counteracted due to reduced anti-oxidative capacity, thereby leading to damage to the central nervous system. It has been reported previously that myeloperoxidase, produced as a result of oxidative stress, increases to threefold higher levels after running a marathon^45,46^.

Consequently, it is important to ensure the optimal amount of physical activity and antioxidant intake in older age, since the ability of endogenous antioxidant production during this phase of life is reduced^47^. The American College of Sports Medicine clearly states that an optimal dosage of sport and other physical activities is essential in older age^47^.

This pioneer longitudinal investigation, which studied the effect of high intensity endurance training on cognitive performance in a later age, comes with a few limitations. First, our sample size is comparably moderate, and the portion of female participants was rather low. Therefore, we could not compare variables between male and female participants. Secondly, we did not investigate daytime activities including gardening, walking, reading, and moderate sports. Therefore, the control group cannot be considered as absolutely sedentary, but as non-athletic. Furthermore, we did not aim to investigate any social effects on cognition. Thus, further studies should be performed focusing on the mutual effect of socioeconomic and physical activity on cognition. Moreover, a more detailed quantification of the control cohort’s actual physical activity level might increase the validity of future investigations.

To conclude, our study provided novel evidence that high intensity endurance training might not be supportive of maintaining cognitive function in the elderly. Future studies will be required to determine whether aged athletes engaged in team sports (rather than in individual endurance sports) will perform better in tests for cognitive functions. Nevertheless, our results suggest that elderly endurance athletes enjoy greater subjective health and well-being.

## Methods

### Study design

In 2008, 136 individuals aged 60 years or older were screened for participation in the Vienna Marathon Study APSOEM (Austrian Prospective Cohort Study in Cognitive Function of Elderly Marathon-runners, clinicaltrials.gov NC M01045031, which was designed as a prospective cohort study. Baseline data were collected in 2008^18,19,35,46^, the first follow-up examinations took place in 2012^48,49^.

The protocol, as well as related amendments, were reviewed and approved by the local ethics committee of the Medical University of Vienna (EK № 401/2005). All medical procedures followed institutional guidelines. All participants gave written informed consent before participation in the study.

### Participants

At baseline, 56 athletes and 58 controls were enrolled. During the follow-up period, 15 participants dropped out due to various reasons (quit physical activity, refused to participate or died). Hence, a total of 99 participants (50 athletes, 49 controls) could be re-evaluated in 2012. The applied inclusion and exclusion criteria are described below. For control participants, which were recruited via personal contact, as well as via newspaper advertisements, the same inclusion and exclusion criteria applied, except for the necessity to have participated in a marathon and the minimum weekly amount of physical training. Instead, control participants had to specify that they did not undergo any strenuous endurance training on a regular basis. Selection of controls was carried out in a frequency-matching process, where sex, age, and years of education served as matching criteria.

### Inclusion criteria

Subjects aged ≥60 in the high intensity endurance training group had to participate in one of the following competitions during the previous three years: Wachau half marathon (21.5 km), Vienna City Marathon (43 km), Carinthian marathon (180 km). Their current amount of physical activity per week must not have been less than 2 hours of intensive training.

### Exclusion criteria

Exposure to neurotoxic substances (queried by occupation anamnesis), a first language other than German (since the neuropsychological test battery was available only in German), the presence of clinically manifest diseases affecting the central nervous system (e.g., ischemia, dementia), cardiovascular diseases or depression or chronic alcoholism and unwillingness to participate in the study led to exclusion.

### Medical Evaluation

Participants underwent a detailed medical check-up including anamnesis, physical examinations and a physical performance test (treadmill test). Moreover, blood was drawn for assessment of hematological, biomedical, genetic, and immunological parameters. Additional blood samples were taken and stored at the MedUni Wien Biobank for subsequent analyses^50^.

### Assessment of cognitive function

Cognitive function was assessed by the German version of a neuropsychological test battery developed by the Consortium to Establish a Registry for Alzheimer’s disease (CERAD). The CERAD Plus test battery contains 15 subtests: Verbal Fluency, Boston Naming Test, Wordlist Encoding, Wordlist Recall, Word list recognition, Constructional Practise and recall, Intrusions, Word list savings, Figure savings, Trail Making Test A, B and BA, S-words and Mini-Mental State Examination. It was mainly designed for the evaluation of executive, frontal and subcortical function^51,52^. All psychological tests were conducted by trained and certified psychologist.

### Health-related quality of life (SF-36)

Additionally, individuals self-assessed their health-related quality of life and well-being status by the Short Form health survey SF-36^53^.

### Treadmill test

Relative physical performance was calculated as the individual maximum workload achieved given as a percentage of the final wattage relative to tabulated sex, age and body surface-specific performance references^54^. The workload was raised every two minutes in steps of 25 W, starting with 25 W and move on until exhaustion. This test is done under monitored electro-cardiograph and blood pressure. The individual physical working capacity (PWC) was calculated as the maximal individual power (WATTmax) in per cent of a reference value (WATTref): PWCind = 100 × WATTmax/WATTref.

### Statistical analysis

Statistical analyses were performed using SPSS 23 (IBM Corp., NY, USA) and Stata 13.0 (StataCorp TX, USA). Results are reported as means and standard deviations (SD). General linear models with repeated measurements design were applied to assess differences between baseline and follow-up (within-subject factor), between groups, or the interaction between time and group (i.e., whether there are differences in time trend between groups). Individuals’ responses to the question about the frequency of conversation with others were used as a covariate to control for possible effects of social communication in the analyses of cognitive performance. P-values were corrected for multiple testing according to Benjamini and Hochberg and given as p_BH_. For all statistical tests, p-values below 0.05 were considered significant.

## Data Availability

Raw data will be available for interested researchers upon request from the corresponding author.
